# Application of three statistical models for predicting the risk of diabetes

**DOI:** 10.1186/s12902-019-0456-2

**Published:** 2019-11-26

**Authors:** Siyu Liu, Yue Gao, Yuhang Shen, Min Zhang, Jingjing Li, Pinghui Sun

**Affiliations:** Epidemiology and Statistics, School of Public Health, Jilin University, Changchun, 130021 China

**Keywords:** Type 2 diabetes, BP neural networks, Logistic regressive model, Decision tree model

## Abstract

**Background:**

At present, the proportion of undiagnosed diabetes in Chinese adults is as high as 15.5%. People with diabetes who are not treated and controlled in time may have various complications, such as cardiovascular and cerebrovascular diseases and diabetic foot disorders, which not only seriously affect the quality of life of people with diabetes but also impose a heavy burden on families and society. Therefore, prevention and control of type 2 diabetes is of great significance.

**Methods:**

We constructed a logistic regression model, a neural network model and a decision tree model to analyse the risk factors for type 2 diabetes and then compared the prediction accuracy of the different models by calculating the area under the relative operating characteristic (ROC) curve and back-inputting the data into the model.

**Results:**

The prevalence of type 2 diabetes in 4177 subjects who were not diagnosed with type 2 diabetes was 9.31%. The most influential factors associated with type 2 diabetes were triglyceride (TG) ≥ 1.17 mmol/L (odds ratio (OR) =2.233), age ≥ 70 years (OR = 1.734), hypertension (OR = 1.703), alcohol consumption (OR = 1.674), and total cholesterol≥5.2 mmol/L (TC) (OR = 1.463). The prediction accuracies of the three prediction models were 90.8, 91.2, and 90.7%, respectively, and the areas under curve (AUCs) were 0.711, 0.780, and 0.698, respectively. The differences in the AUCs after back propagation (BP) of the neural network model, logistic regression model and decision tree model were statistically significant (*P* < 0.05).

**Conclusion:**

BP neural networks have a higher predictive power for identifying the associated risk factors of type 2 diabetes than the other two models, but it is necessary to select a suitable model for specific situations.

## Background

Diabetes mellitus (DM) is a metabolic disease characterized by elevated blood glucose. All over the world, the prevalence of diabetes is on the rise. According to a WHO report, there have been more than 36 million deaths due to chronic non-communicable diseases worldwide, and diabetes ranked fourth, accounting for 3% in 2008 [1]. By 2014, the number of people with diabetes worldwide reached 422 million, accounting for 8.5% of the total population [2]. In recent years, the proportion of deaths due to diabetes has gradually increased as a proportion of all deaths due to chronic diseases. According to existing diabetes data and trends, it is estimated that the number of people with diabetes worldwide will reach 366 million by 2030 [3]. At present, the proportion of the potential population with diabetes in Chinese adults is as high as 15.5, and 60.7% of these individuals had not previously received a diagnosis of prediabetes [4].

Diabetes is a chronic, lifelong disease. Diabetes is one of the main chronic non-communicable diseases and can cause various complications, such as hypertension, coronary heart disease, diabetic nephropathy and diabetic foot [5]. Diabetes has become one of the main sources of global disease burden [6]. At present, diagnosis of diabetes is mainly based on a doctor’s diagnosis and laboratory examination. In some cases, diabetes complications can be avoided when a person with diabetes is diagnosed early, treated and maintains tight control of their blood sugar levels. However, due to the lack of early diagnosis and screening techniques, some diabetic patients develop into advanced stages when they are diagnosed [4], which causes serious consequences, and some have even died [7]. Diabetes consumes a large amount of medical resources. How to solve the contradiction between the uneven distribution of medical resources and the rapid growth of medical expenses is a problem.

Prevention-based control is needed for type 2 diabetes. Studies have shown that community intervention is a cost-effective measure to reduce cardiovascular morbidity throughout the world [8, 9] and the risk of diabetes in individuals. An assessment can screen out high-risk groups with diabetes and reduce the incidence and mortality of diabetes by targeting intervention in high-risk groups. Knowler et al. conducted a follow-up for an average of 2.8 years and concluded that lifestyle interventions reduced the incidence of pre-diabetic patients by 58% [10]. The Finnish Diabetes Prevention Study reported that obese pre-diabetes patients with weight loss of more than 2.5% within one year had an incidence of diabetes of approximately 2%, and patients with a 2.5% weight gain had an incidence of diabetes of approximately 8%. Perreault et al. reported that reverting to normal glucose levels can reduce the risk of diabetes in the future by 56% in pre-diabetes patients [11].

In recent years, most of the methods for performing a disease risk assessment involve data mining technology. Data mining is a new widely used method in the medical field for disease diagnosis, prognosis, medical expense management. Wang C. et al. used a neural network to identify those at high risk of T2DM based on demographic, lifestyle and anthropometric data [12]. Kang S. et al. used a neural network to make a personalized prediction of drug efficacy for diabetes treatment [13]. Kim SY at al.(2011) used an artificial neural network to establish a predictive model of pre-operative advanced prostate cancer, providing a basis for clinical decision-making [14]. Hon-Yi Shi et al. used artificial neural network and logistic regression models to predict in-hospital mortality after traumatic brain injury surgery [15].

Logistic regression models are nonlinear probability models that are typically used to identify disease risk factors and predict the risk of occurrence. These models are suitable for performing regression analyses of dependent variables as categorical variables. The factors that influence diabetes have been widely used in logistic regression analyses in the past. The BP neural network is a multi-layer, feedforward neural network trained by an error back propagation algorithm. The BP neural network is the most widely used neural network technique and uses computing power to simulate the information transmission process of an animal neural network. Perez Acadia used an artificial neural network to establish a predictive model of hyperglycaemia in diabetic patients [16]; Zarkogianni K used an artificial neural network to establish a query system for insulin injection in people with diabetes [17]. A decision tree model is a tree-like process in which each node is a split attribute that can be intuitively seen from the decision tree. The decision tree model has the advantages of being fast, easy to understand, and able to process large amounts of data. The decision tree model has been widely used in various medical fields in recent years [18]

Data mining is widely used in the medical field, although in recent years, studies have used using data mining techniques to study predictive models, such as logistic regression analysis, decision trees, artificial neural network algorithms, and others. Most of the studies use a single model and rarely use multiple models to conduct comparative research [4, 12, 13]. Because each method has its own advantages and disadvantages, it is necessary to compare different models to identify the optimal mathematical model for predicting type 2 diabetes. This paper combines a logistic regression model, a BP neural network model and a decision tree model to analyse factors affecting diabetes and explore the most suitable model for predicting the risk of type 2 diabetes in the Chinese population. In addition, this study was based on adults in Northeast China, inhabitants of this area have unique life style than others, including higher drinking rate and high salt and oil diet. Due to different lifestyles, local residents may have different causes of type 2 diabetes than other regions.

## Methods

### Participants

This study was conducted in 2018. Participants were those who resided in the 5 monitoring areas of Jilin Province for 6 months or more within 12 months prior to the survey and were 18 years old or older. According to the multi-stage stratified cluster sampling method, 5 counties (cities) were selected as monitoring points in each stage, 4 towns (streets) were selected in each monitoring point, and 3 villages (residential committees) were selected in each township. Each village drew 50 families, and each family drew a resident over 18 years old to include. To control lost calls, this study adopted homologous population replacement for lost participants and controlled the replacement rate of the surveyed households to not exceed 10.0%. The exclusion criteria were (1) lack of diabetes-related laboratory tests data and questionnaire data or (2) self-report of a type 2 diabetes diagnosis on the questionnaire. A total of 4689 cases were recovered, of which 4177 were valid.

## Research methods

The survey included three parts: a questionnaire, physical examinations, and laboratory tests.

### Questionnaire survey

The questionnaire survey was conducted in accordance with the China Chronic Disease Surveillance Questionnaire Survey Procedure and was conducted face-to-face by investigators with unified training [19]. The questionnaire included smoking, alcohol, diet, and physical activity. Smoking meant that at least one tobacco product was consumed every day and there was a history of smoking for ≥6 months consecutively. Drinking was consumption of any type of alcohol at least once a week and a history of drinking for ≥6 months.

### Physical examination

Height, weight, waist circumference, and blood pressure were measured by two uniformly trained surveyors, and body mass index BMI = weight (kg) / height ^2^ (m^2^) [20]. The accuracy of the height measurement tools is 0.1 cm. The accuracy of the weight measurement tools is 0.1 kg. The accuracy of the waist measurement tools is 0.1 cm. Blood pressure was measured using a HEM-7200 electronic sphygmomanometer produced by Omron (Dalian) Co Ltd. BMI < 27.9 kg/m^2^ was normal and overweight and BMI ≥ 28.0 kg/m^2^ was obese [21]. A male waist circumference > 90 cm and a female waist circumference > 80 cm was indicative of abdominal obesity [22, 23]. Systolic blood pressure ≥ 140 mmHg (1 mmHg without antihypertensive drug) =0.1333 kPa and diastolic blood pressure ≥ 90 mmHg or having been diagnosed with hypertension by a township hospitals in the past 2 weeks were considered high blood pressure [24].

### Laboratory tests

The investigator took 4 ml of fasting venous blood from the surveyed subjects, and after centrifugation and dispensing at the survey site, the samples were stored and transported to the Jilin University School of Basic Medicine laboratory for determination of fasting blood glucose and oral glucose after oral administration of 75 g of anhydrous glucose for 2 h (OGTT-2 h), glycated haemoglobin (HbA1c), cholesterol (TC), triglyceride (TG), low density lipoprotein cholesterol (LDL-C) and high density lipoprotein cholesterol (HDL-C) and other indicators.

Diabetes mellitus was diagnosed if fasting blood glucose was ≥7.0 mmol / L, OGTT - 2 h blood glucose was ≥11.1 mmol / L or HbA1c was ≥6.5% [25]. The participants who were not diagnosed with dyslipidemia and were not taking lipid-lowering drugs. If TC was ≥5.2 mmol / L, then TC was considered increased. If TG was ≥1.17 mmol / L, then TG was considered increased. If LDL - C was ≥3.4 mmol / L, then LDL-C was considered increased. If HDL-C was < 1.04 mmol/L, then HDL-C was considered reduced [26]. In people who have been diagnosed with dyslipidemia in the past, we asked them to answer the supplementary question in the questionnaire, which one of the high triglycerides, high cholesterol, high LDL-c or low HDL-c is diagnosed (If there are multiple indicators of one sample diagnosed as abnormal, it would be included in multiple variables).

### Statistical analysis

We used Epi Data 3.1 software with double-entry data to establish a database and complete a consistency test. IBM SPSS 24.0 statistical software was used for general descriptive analysis, chi-square tests and establishing the logistic regression model, the BP neural network model and the decision tree model. Of the 4177 participants in this study, 70% of subjects (n1 = 2924) were randomly selected to provide a training data set and 30% of subjects (n2 = 1253) were selected to provide a validation data set for the logistic regression model and the decision tree model, 274 (9.37%) and 115(9.18%) people with type 2 diabetes fell in each set. For BP neural network model, we extract 1/3 from the training set as the testing set, 193 (9.47%) and 81 (9.24%) people with type 2 diabetes fell in training set and testing set. We used cross validation to verify the model. The logistic regression model required considering the collinearity problem when incorporating variables. We used tolerance values and the variance inflation factor (VIF) to examine collinearity. The criteria values for tolerance and VIF (≤0.10 and ≥ 10, respectively) were sufficient to identify co-linearity and thus be excluded when entering the model. The analysis results were statistically significant at *P* < 0.05.

## Results

### Demographic characteristics

Comparing the prevalence of diabetes in people with different demographic characteristics, the results showed that the prevalence of type 2 diabetes was significantly different among subjects of different genders and ages, and the significance level was set at 0.05. The results are shown in Table 1.

**Table 1:** Comparison of the prevalence of type 2 diabetes in populations with different demographic characteristics (*N* = 4177)

Variables	Levels	Type 2 diabetes	Prevalence (%)	χ^2^	*P*
Gender	Men	184	10.6	5.720	0.017
	Women	205	8.4		
Age	< 70	314	8.5	23.801	< 0.001
	≥70	75	15.3		
Nation	Han nationality	331	9.1	1.781	0.182
	Minority	58	10.9		
Place of residence	Urban	215	9.4	0.042	0.837
	Rural	174	9.2		
Education level	Primary or below	123	9.8	1.646	0.649
	Junior middle school	133	9.4		
	Senior high school	77	8.3		
	College or higher	56	9.7		
Marital status	Married	321	9.2	0.111	0.739
	Other	68	9.6		
Occupation	Physical work	228	9.0	2.639	0.267
	Mental work	70	8.8		
	Retirement or other	91	10.8		

Comparing the prevalence of diabetes in people with different lifestyles, the results showed that the prevalence of type 2 diabetes was statistically significant among subjects with different smoking and drinking statuses, and the significance level was set at 0.05. The results are shown in Table 2

**Table 2:** Comparison of the prevalence of type 2 diabetes in different lifestyle groups (*N* = 4177)

Variables	Levels	Type 2 diabetes	Prevalence (%)	χ^2^	*P*
Smoking	Yes	115	10.9	4.005	0.045
	No	274	8.8		
Drinking	Yes	119	13.4	22.315	< 0.001
	No	270	8.2		

Comparing the prevalence of type 2 diabetes in people with different health statuses, the results showed that the prevalence of type 2 diabetes was statistically significant among subjects with different BMI, abdominal obesity, hypertension, and stroke, and the significance level was set at 0.05. The results are shown in Table 3

**Table 3:** Comparison of the prevalence of type 2 diabetes in different health status groups (*N* = 4177)

Variables	Levels	Type 2 diabetes	Prevalence (%)	χ^2^	*P*
BMI	< 28	281	8.0	41.284	< 0.001
	≥28	108	15.9		
Abdominal obesity	Yes	263	12.3	48.454	< 0.001
	No	126	6.2		
Hypertension	Yes	215	14.7	77.458	< 0.001
	No	174	6.4		
Myocardial infarction	Yes	6	12.0	0.434	0.510
	No	382	9.3		
Stroke	Yes	23	17.8	11.439	0.001
	No	365	9.0		
Chronic obstructive pulmonary diseases	Yes	13	13.5	2.072	0.150
	No	375	9.2		
Asthma	Yes	11	13.4	1.651	0.199
	No	377	9.2		
Cancer	Yes	7	6.7	0.901	0.343
	No	380	9.4		

Comparing the prevalence of type 2 diabetes in people with different health statuses, the results showed that the prevalence of type 2 diabetes was statistically significant among subjects with different TC, TG, LDL-C, and HDL-C, and the significance level was set at 0.05. The results are shown in Table 4

**Table 4:** Comparison of the prevalence of type 2 diabetes in different health status groups (*N* = 4177)

Variables	Levels	Type 2 diabetes	Prevalence (%)	χ^2^	*P*
TC	Normal	210	7.3	46.512	< 0.001
	Above normal	179	13.9		
TG	Normal	168	5.9	32.766	< 0.001
	Above normal	221	16.5		
LDL-C	Normal	333	8.8	10.956	0.001
	Above normal	56	13.9		
HDL-C	Normal	243	7.9	30.077	< 0.001
	Below normal	146	13.7		

#### Logistic regression model

Inclusion of meaningful variables from the univariate analysis in the multivariate logistic analysis showed that 7 variables were statistically significantly associated with the onset of type 2 diabetes: drinking (*P* < 0.001, OR = 1.674), age (*P* < 0.001, OR = 1.734), waist circumference (*P* = 0.006, OR = 1.448), blood pressure (*P* < 0.001, OR = 1.703), TC (*P* = 0.003, OR = 1.463), BMI (*P* = 0.047, OR = 1.321), and TG (P < 0.001, OR = 2.233). See the table for details. As a predictor of the logistic model, the predictive model is: *P* = 1 / (1 + e (3.569–0.515 × drinking - 0.550 × age - 0.370 × abdominal obesity - 0.533 × hypertension - 0.381 × TC - 0.297 × BMI -0.803 × TG)). Among them, P is the predicted probability of the logistic regression model. Between 0 and 1, the closer P is to 1, the greater the probability of developing type 2 diabetes. Substituting the prediction model into the testing data set, with a critical value of 0.5, the results show that the prediction accuracy of the model was 90.8% and the area under the ROC curve was 0.711 (95% Cl: 0.697–0.724). The sensitivity of the ROC curve was 67.8%, and its specificity was 64.7%. The results are shown in Table 5

**Table 5:** Multivariate logistic regression analysis of factors affecting type 2 diabetes (*N* = 4177)

Variables	Comparison	Control	β	SE	Waldχ^2^	*P*	OR	95%C.l. for OR
Gender	Women	Men	0.053	0.143	0.135	0.713	1.054	0.796–1.396
Smoking	Yes	No	0.060	0.141	0.180	0.671	1.062	0.805–1.401
Drinking	Yes	No	0.515	0.147	12.305	< 0.001	1.674	1.255–2.232
Age	≥70	< 70	0.550	0.151	13.236	< 0.001	1.734	1.289–2.332
Abdominal Obesity	Yes	No	0.370	0.135	7.507	0.006	1.448	1.111–1.887
Hypertension	Yes	No	0.533	0.117	20.778	< 0.001	1.703	1.355–2.142
Cerebral Stroke	Yes	No	0.327	0.254	1.655	0.198	1.387	0.843–2.281
BMI	28~	< 28	0.279	0.140	3.952	0.047	1.321	1.004–1.739
TG	Above normal	Normal	0.803	0.129	38.869	< 0.001	2.233	1.735–2.875
TC	Above normal	Normal	0.381	0.129	8.693	0.003	1.463	1.136–1.885
LDL-C	Above normal	Normal	−0.091	0.18	0.254	0.615	0.913	0.641–1.301
HDL-C	Below normal	Normal	0.116	0.131	0.782	0.376	1.123	0.868–1.453
Constant			−3.569	0.165	467.666	0	0.028	

#### BP neural network model

The 13 variables that were significant by the chi-square test were included in the neural network model. That is, 13 units were established in the input layer. The number of hidden layers can be 1 or 2. The hidden layer activation function is a hyperbolic tangent, the input layer activation function is the softmax, and the output layer has two units. First, when the hidden layer was 1, the area under the ROC curve of the model with a different number of hidden layer nodes was discussed. The results are shown in Table 6. When the number of hidden layer nodes was 5, the area under the ROC curve was the largest, 0.780 (95% Cl: 0.767–0.792); the sensitivity was 72.94%; and the specificity was 72.42%. The results are shown in Table 6

**Table 6:** Area under the ROC curve of different hidden layer nodes with one hidden layer in the BP neural network model (*N* = 4177)

Number of hidden layer nodes	The area under ROC curve (95%Cl)	Z	P
1	0.725(0.711–0.738)	18.109	< 0.0001
2	0.737(0.723–0.750)	19.845	< 0.0001
3	0.746(0.732–0.759)	19.379	< 0.0001
4	0.755(0.741–0.768)	21.893	< 0.0001
5	0.780(0.767–0.792)	24.931	< 0.0001
6	0.778(0.765–0.790)	23.229	< 0.0001
7	0.765(0.752–0.778)	22.774	< 0.0001
8	0.754(0.741–0.767)	22.532	< 0.0001
9	0.735(0.721–0.748)	19.015	< 0.0001
10	0.728(0.714–0.741)	19.158	< 0.0001

The area under the ROC curve of different hidden layer nodes with two hidden layers in the BP neural network model is shown in Table 7

**Table 7:** Area under ROC curve of different hidden layer nodes with two hidden layers in the BP neural network model (*N* = 4177)

Number of first hidden layer nodes	Number of second hidden layer nodes	The area under ROC curve (95%Cl)	Z	P
4	1	0.724(0.710–0.737)	18.900	< 0.0001
4	2	0.736(0.722–0.747)	18.895	< 0.0001
4	3	0.745(0.731–0.758)	20.495	< 0.0001
4	4	0.748(0.734–0.761)	19.944	< 0.0001
5	1	0.752(0.739–0.765)	19.326	< 0.0001
5	2	0.760(0.746–0.773)	23.355	< 0.0001
5	3	0.753(0.740–0.766)	19.337	< 0.0001
5	4	0.750(0.737–0.763)	20.810	< 0.0001
6	1	0.741(0.725–0.752)	19.730	< 0.0001
6	2	0.742(0.726–0.753)	20.365	< 0.0001
6	3	0.740(0.724–0.751)	19.095	< 0.0001
6	4	0.737(0.721–0.748)	18.460	< 0.0001

Finally, the BP neural network with 1 hidden layer and 5 hidden layer nodes was taken as the final prediction model. The BP neural network ranks the importance of predictor variables for different predictors, and the details are shown in Table 8

**Table 8:** Importance of variables for predicting type 2 diabetes (*N* = 4177)

Predictor	Importance	The Importance Of
TG	0.167	100.0%
Drinking	0.153	91.4%
Age	0.125	74.9%
Hypertension	0.098	58.8%
TC	0.084	50.4%
Cerebral Stroke	0.084	50.4%
BMI	0.068	40.8%
Abdominal Obesity	0.056	33.3%
Smoking	0.051	30.7%
LDL-C	0.040	24.0%
Gender	0.039	23.3%
HDL-C	0.033	19.9%

Dividing the predictor importance indicator by the maximum indicator value yields a normalized predictor importance order. The results showed that the top five variables were TG (100.0%), alcohol consumption (91.4%), age (74.9%), hypertension (58.8%), and TC (50.4%). Using the BP neural network model for prediction, with 0.5 as the predicted quasi-probability boundary value, the prediction accuracy of the model was 91.3% and the area under the ROC curve was 0.781 (95% Cl: 0.768–0.794). The results are shown in Table 7

#### Decision tree model

**Fig. 1:**
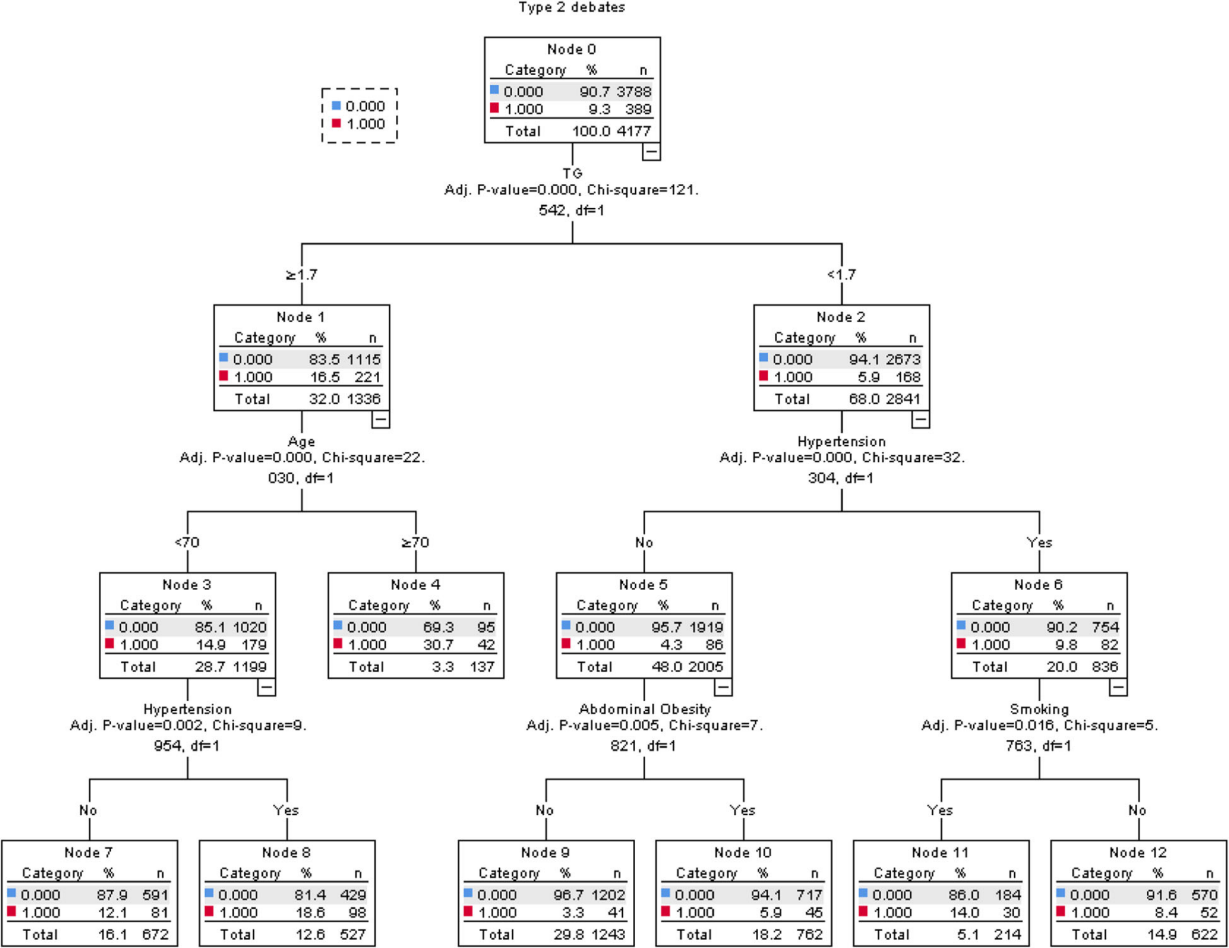
Decision tree model diagram of type 2 diabetes

The decision tree for factors affecting the prevalence of type 2 diabetes is shown in Fig. 1. It can be seen from the figure that the first layer is TG, indicating that TG had the strongest correlation with type 2 diabetes and that the risk of type 2 diabetes was higher in people with higher TG than normal. The remaining variables were hypertension, age, smoking, and abdominal obesity. The accuracy of the prediction model was 90.7%, and the area under the ROC curve was 0.698 (95% CI: 0.684–0.712). The sensitivity of the curve was 64.5%, and the specificity was 65.7%. The results are shown in Fig. 1

#### Model comparison

The prediction accuracy of the three models and the area under the ROC curves are shown in Table 8. The area under the ROC curve is ranked from BP neural network model (0.780) to logistic regression model (0.711) to decision tree model (0.698). Three models’ AIC are 293.178, 281.895, 343.877. The results are shown in Table 9

**Table 9:** Areas under the ROC curves for the three models

Predictive model	Accuracy	AUC	SE	95%Cl	*P*	AIC
Logistic Regression model	90.8	0.711	0.0137	0.697–0.724	< 0.0001	293.178
BP Neural network model	91.2	0.780	0.0128	0.767–0.792	< 0.0001	281.895
Decision tree model	90.7	0.698	0.0136	0.684–0.712	< 0.0001	343.877

The areas under the ROC of different models are shown in Table 10. The results showed that the difference between the ROC area values of the BP neural network model and the logistic regression model was statistically significant (*P* < 0.001), the difference between the ROC area values of the BP neural network model and the decision tree model was statistically significant (P < 0.001), but the difference between the ROC area values of the logistic regression model and the decision tree model was not statistically significant (*P* = 0.0711 ≥ 0.05).

**Table 10:** Comparison of the area under the ROC of the three models

Predictive model	SE	Z	P
BP Neural network model vs Logistic Regression model	0.0111	6.210	< 0.001
Logistic Regression model vs Decision tree model	0.0100	1.310	0.190
BP Neural network model vs Decision tree model	0.0108	7.649	< 0.001

**Fig. 2:**
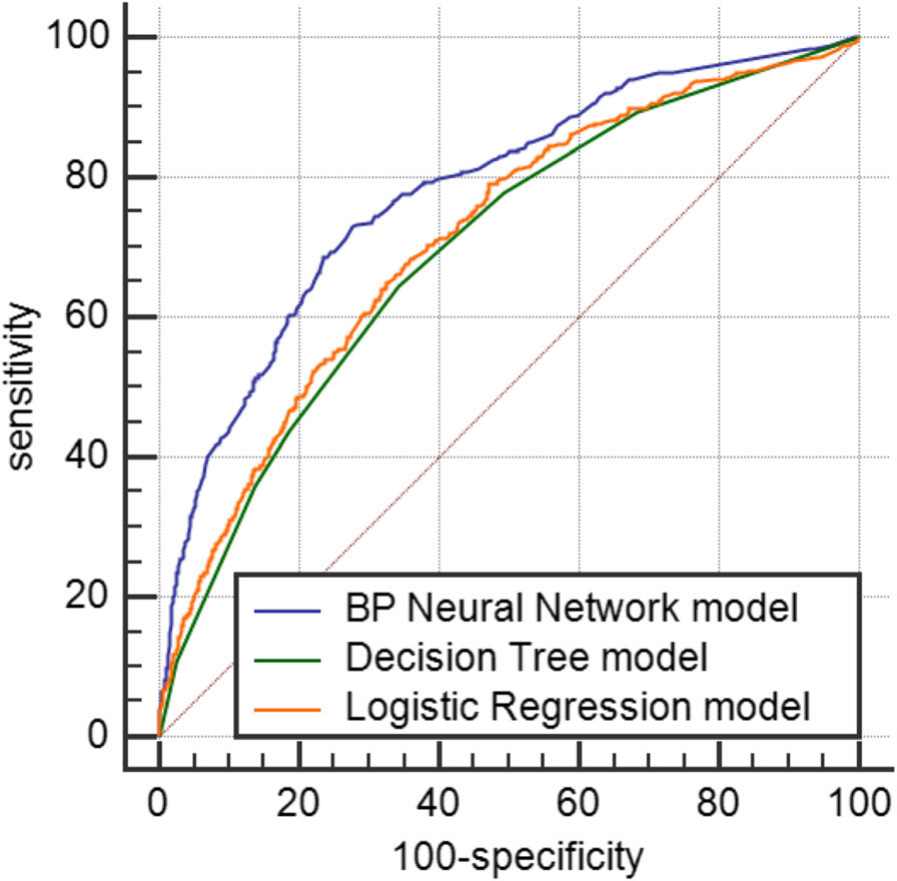
ROC curves of the three prediction models

Figure 2 shows the ROC curves of the three models. The ordinate is sensitivity, reflecting the ability of the model to correctly identify a patient. The abscissa is 100-specific, reflecting the ability of the model to misjudge a patient. The larger the ordinate of the model, the smaller the abscissa. That is, the larger the area under the ROC curve and the closer to 1, the better the diagnostic effect of the model. As shown in Fig. 2, the BP neural network model has the largest area under the ROC curve, indicating that it has the best diagnostic value compared with the other two models

## Discussion

### Analysis of factors influencing type 2 diabetes

Multivariate logistic regression model, BP neural network model, and decision tree model predictions showed that the main risk factors for type 2 diabetes included TG, age, blood pressure, drinking, TC, waist circumference, and BMI. Among them, the most influential factors associated with the onset of type 2 diabetes were TG (OR = 2.233), age (OR = 1.734), hypertension (OR = 1.703), alcohol consumption (OR = 1.674), and TC (OR = 1.463).

Hypertension, high triglycerides, and high total cholesterol were risk factors for diabetes and cardiovascular disease that have been previously confirmed [27]. This study showed that people aged 70 years and older were more susceptible to type 2 diabetes than those below 70. Most studies have presented the same result [28]. As age increases, collagen and elastin in blood vessels gradually decreases, the blood vessel wall becomes hard and brittle, blood pressure and blood lipids rise, and high density lipoprotein cholesterol decreases. The hardening and aging of blood vessels and the change in the normal function of the vascular wall caused by inflammatory reactions and adipose tissue can also affect the expression of intracellular protein kinases, regulating the expression of inflammatory genes [29–31], affecting the normal function of islet β cells and reducing insulin secretion. Hypertension often coexists with diabetes [32], which may have a common risk factor, such as obesity.

This study suggests drinking is a risk factor for type 2 diabetes. Studies have shown that [33] moderate drinking can improve insulin sensitivity, increase high-density lipoprotein cholesterol and adiponectin levels, and reduce inflammation, but heavy drinking increases energy intake and causes obesity, impairs liver function, and increases the pancreatic burden, which accelerate the progression of diabetes. Therefore, to control the prevalence of diabetes and its complications, smoking and drinking should be an important target for prevention and control.

Abdominal obesity and BMI > 28 are both considered obese and have been proven to be important risk factors for many chronic, non-communicable diseases. Adipose tissue can release a large number of inflammatory cytokines and biologically active regulatory factors that aggravate the body’s oxidation. The agonistic reaction produces an inflammatory reaction, affects the secretion and regulation of insulin, and causes the body to produce insulin resistance, which affects the stability of blood sugar levels.

### Diabetes risk prediction

#### BP neural network model

The results of this study showed that when predicting the risk of type 2 diabetes, the prediction accuracy of the BP neural network model was 91.2% and the area under the ROC curve was 0.780. The BP neural network model was better than the logistic regression model and the decision tree model both in accuracy and the area under the ROC curve, and the difference between them was statistically significant (*P* < 0.05).

Compared with the logistic regression model, the BP neural network model is not affected by the interactions between variables and has nonlinear mapping abilities, self-learning and self-adaptive abilities, generalization abilities, and fault tolerance. It can handle complexities better than other models. The data have been widely used in the medical field. There have been many studies on BP neural networks in the past. In Shi HY et al. [15], artificial neural network and logistic regression models were used together to predict in-hospital mortality after traumatic brain injury surgery. The results showed that the artificial neural network was a better prediction model in terms of accuracy and area under the ROC curve. Li Lixia et al. [34] used a logistic regression model and a BP neural network model to predict liver cancer and also concluded that the BP neural network model was superior to the logistic regression model. When the BP neural network model was established in this paper, the area under the ROC curve with 1 layer and 2 layers of hidden layers and different hidden layer nodes was selected. The comparison result was that when the hidden layer was set to 1, the number of hidden layer nodes was 5. The area under the ROC curve was the largest, and the prediction ability was the best. Previous studies have shown that BP neural networks with a hidden layer of 1 better predict ischemic stroke [35]. However, the BP neural network model also has many shortcomings, such as an (1) “overfitting” phenomenon. If the BP neural network is too detailed from the training sample, the learned model does not correctly reflect the law applied in the sample. Therefore, grasping the degree of learning and the correct generation of rules is essential. (2) Determination of the number of hidden layers is also a shortcoming. There is no theoretical guidance for the choice of the number of layers and the number of cells in the hidden layer of the network, which are generally determined by experience or after repeated experiments. Most of the existing research results show that a hidden BP neural network can reflect the data rules and characteristics well. When the BP neural network model was established in this paper, the area under the ROC curve when the number of hidden nodes was 1 or 2 was compared. The results showed that the prediction was better when the number of hidden layers was 1. (3) The BP neural network cannot judge whether the variable is a protective factor or a risk factor. (4) The model cannot perform hypothesis testing or medical interpretation of the weighted coefficients.

#### Decision tree model

The decision tree model had a short computation time, and the results were simple and intuitive to display in a tree. The classification power of the results was more accurate. However, when the classification increased, it affected the prediction results [36]. The decision tree model can only process categorical variables. Continuous variables cannot be included. Moreover, a common shortcoming of the BP neural network model and decision tree model is that the direction of the variable cannot be explained. The research factor cannot be judged to be a risk factor or a protective factor, whereas the logistic regression model can explain the direction of the variable well. The area under the ROC curve of the decision tree model in this paper was the smallest of the three prediction models, and the difference between the BP neural network model and the logistic regression model was statistically significant. Some scholars have compared decision trees with other statistical models. Li Xianwen et al. [37] found that the prediction of a logistic regression model was better than that of a decision tree model in a study of health literacy in hypertensive patients, in agreement with the results of this paper.

Rapid and effective prediction of the risk of type 2 diabetes can allow for preventative actions to be taken by members of high-risk groups. The results of this study showed that the BP neural network model was a good predictive model for type 2 diabetes, but for practical applications, the logistic regression model can explain the variables and results more intuitively. The BP neural network model and the decision tree model lack the ability to interpret results. Therefore, for practical applications, it is necessary to combine the advantages and disadvantages of each model and select the appropriate model to obtain the highest value in practice.

## Conclusions

BP neural networks have a higher predictive power for identifying the associated risk factors of type 2 diabetes than Logistic regression model and decision tree model, but it is necessary to select a suitable model for specific situations.

## Data Availability

The datasets used and analysed during the current study are available from the corresponding author on reasonable request.

## Abbreviations

ROC: Relative operating characteristic curve
TG: Triglyceride
TC: Total cholesterol
OR: Odds ratio
AUC: Areas under curve
DM: Diabetes mellitus
BP: Back propagation
OGTT-2 h: Anhydrous glucose for 2 h
HbA1c: Glycated haemoglobin
LDL-C: Low density lipoprotein cholesterol
HDL-C: High density lipoprotein cholesterol
VIF: Variance inflation factor
